# Early Phase Gingival Wound Healing Following Low-Level Er:YAG Laser Irradiation: In Vitro and In Vivo Studies

**DOI:** 10.3390/dj14030138

**Published:** 2026-03-02

**Authors:** Lu Chen, Koji Mizutani, Natsumi Saito, Bruna Akinaga Moreira, Daisuke Kido, Takanori Iwata, Akira Aoki

**Affiliations:** 1 Department of Periodontology, Graduate School of Medical and Dental Sciences, Institute of Science Tokyo (Science Tokyo), Bunkyo-ku, Tokyo 113-8549, Japanaoki.a.3ef4@m.isct.ac.jp (A.A.); 2 Department of General Dentistry, Graduate School of Medical and Dental Sciences, Institute of Science Tokyo Hospital, Bunkyo-ku, Tokyo 113-8549, Japan

**Keywords:** angiogenesis, endothelial cells, Er:YAG laser, gingiva, low-level laser irradiation, photobiomodulation, wound healing

## Abstract

**Background:** Low-level laser irradiation (LLLI) can promote wound healing. However, the biological effects of the erbium-doped yttrium aluminum garnet (Er:YAG) laser on gingival wound healing remain unclear. **Objectives:** To assess the effects of low-level Er:YAG laser irradiation on endothelial cell activity in vitro and on early phase gingival wound healing in vivo. **Methods**: In vitro, human umbilical vein endothelial cells were irradiated with a low-level Er:YAG laser (30 mJ/pulse, 10 Hz, 20 and 30 s, defocused, no water spray) and assessed for viability, cytotoxicity, and migration. Standardized bilateral wounds (4 × 1 mm) were created in the palatal gingiva of 14 male mice using a scalpel and curette. The wounds were irradiated for 20 s under the same irradiation settings, using a contact tip (diameter 800 μm) to induce superficial blood surface coagulation, while contralateral sites were assigned to controls in a split-mouth design. Postoperative wound area and mRNA expression of IL-6, TNF-α, VEGF, FGF-2, and TGF-β1 were analyzed after 48 h. **Results:** In vitro, LLLI significantly enhanced cell proliferation with/without increasing cytotoxicity. In the wound healing assay, the LLLI significantly promoted cell migration compared with the control. In vivo, the reduction in residual wound area in the laser group was comparable to that in the control group. IL-6 and TNF-α expressions were significantly downregulated, whereas VEGF was significantly upregulated in the laser group. **Conclusions:** Low-level Er:YAG laser irradiation enhances anti-inflammatory and pro-angiogenic effects, suggesting its potential in promoting gingival wound healing.

## 1. Introduction

Low-level laser irradiation (LLLI) or photobiomodulation (PBM) has recently emerged as a non-invasive adjunctive therapeutic approach for enhancing wound healing and tissue repair across a wide range of medical and dental applications [1,2]. Unlike high-energy laser ablation, LLLI employs low-power, low-thermal laser irradiation to modulate cellular behavior through photochemical and photophysical interactions, leading to alterations in mitochondrial activity, intracellular signaling, and gene expression [3]. In dentistry, LLLI can reduce inflammation and pain while promoting soft and hard tissue healing in periodontal, implant, and oral surgical procedures [1,2].

Gingival wound healing represents a particularly challenging biological process due to the unique oral environment. Gingival tissues are constantly subjected to mechanical stress during mastication and speech, as well as persistent microbial exposure from the oral biofilm [4,5]. Successful gingival wound repair requires precise temporal coordination of early inflammatory responses and angiogenesis for supporting granulation tissue formation and tissue maturation [6,7]. Excessive or prolonged inflammation, characterized by sustained expression of pro-inflammatory cytokines, such as interleukin-6 (IL-6) and tumor necrosis factor-α (TNF-α), is closely associated with impaired wound healing and chronic wound development [7].

Angiogenesis, which is a central event during the early proliferative phase of wound healing, is essential for supplying oxygen, nutrients, and bioactive molecules to regenerating tissues [8]. Vascular endothelial growth factor (VEGF) plays a pivotal role in this process by promoting endothelial cell proliferation, migration, and neovascular formation [9,10]. Therefore, therapeutic strategies that can simultaneously modulate inflammation and enhance angiogenesis are highly desirable for optimizing gingival wound healing outcomes.

Among dental laser systems, the erbium-doped yttrium aluminum garnet (Er:YAG) laser, with a wavelength of 2.94 μm, has been widely adopted in periodontal and oral surgical therapy, owing to its strong absorption by water and hydroxyapatite, allowing precise ablation with minimal thermal damage [11]. Clinically, Er:YAG lasers have demonstrated effectiveness in minimally invasive subgingival calculus removal and periodontal debridement [12,13], as well as demonstrating bactericidal and decontamination effects on periodontopathic microorganisms [14,15]. Furthermore, comprehensive reviews have highlighted favorable periodontal and peri-implant wound healing responses following Er:YAG laser-assisted therapy [16,17].

Beyond their ablative applications, increasing evidence indicates that Er:YAG lasers operated at sub-ablative energy levels can exert biological effects consistent with LLLI. In vitro studies have demonstrated that low-level Er:YAG laser irradiation enhances the proliferation and metabolic activity of human gingival fibroblasts, accompanied by differential protein expression related to wound repair [18,19]. Additional investigations have shown that low-level Er:YAG irradiation activates intracellular signaling pathways, such as mitogen-activated protein kinase/extracellular signal-regulated kinase (MAPK/ERK), which promote osteoblastic proliferation and calcification [20,21]. These findings suggest that low-level Er:YAG laser irradiation may influence cellular behavior beyond tissue removal, potentially contributing to regenerative processes.

Recent studies suggest that LLLI using Er:YAG lasers can modulate inflammatory responses. Ng et al. [22] reported that low-level Er:YAG laser irradiation suppressed the expression of pro-inflammatory cytokines and inflammasome activation in human periodontal ligament fibroblasts stimulated with *Porphyromonas gingivalis* lipopolysaccharide. These observations are consistent with the broader literature on LLLI, indicating that laser irradiation can attenuate excessive inflammatory signaling, partly through regulation of the nuclear factor-κB (NF-κB)-related pathways [23,24].

Importantly, low-level Er:YAG laser irradiation may also enhance angiogenic signaling. Takemura et al. [25] demonstrated that LLLI using an Er:YAG laser significantly upregulated VEGF expression and promoted periodontal tissue healing in in vitro and in vivo models. Most LLLI-related angiogenesis studies have focused on diode or Nd:YAG lasers and related clinical observations using non-ablative Nd:YAG laser therapy in gingival tissues have also been reported [26], leaving the biological effects of Er:YAG-based LLLI insufficiently characterized [27].

Therefore, this study aimed to investigate the biological effects of low-level Er:YAG laser irradiation applied as LLLI on early gingival wound healing using a combined in vitro and in vivo approach. Specifically, we evaluated the effects of Er:YAG laser-based LLLI on endothelial cell proliferation, migration, and cytotoxicity in vitro, and assessed its influence on early phase gingival wound healing and the expression of key inflammatory and angiogenic markers in a murine model. By clarifying the cellular and molecular responses induced by Er:YAG laser–based LLLI, this study sought to contribute to the understanding of biological processes associated with its potential role as an adjunctive therapeutic modality for enhancing gingival soft tissue healing.

## 2. Materials and Methods

### 2.1. Er:YAG Laser Apparatus

An Er:YAG laser device (Adverl SH; Morita, Japan) was used. The laser system employs a contact tip and handpiece system. LLLI was performed in a defocused mode without water spray at 10 Hz, 30 mJ/pulse, pulse width 122 μs, and peak power 508 W, for 20 or 30 s. The 20 s irradiation was selected based on previous studies [25] and/or clinically relevant parameters. The 30 s irradiation was included as a higher irradiation dose to examine the dose-dependent biological responses. The actual energy level was measured using an energy meter (EnergyMax™ Sensors; Coherent, Santa Clara, CA, USA) before each session to ensure a stable delivery.

### 2.2. In Vitro Study

#### 2.2.1. In Vitro Cell Culture and Laser Irradiation

Human umbilical vein endothelial cells (HUVECs; Lonza, Basel, Switzerland) were used. The cells were cultured in endothelial growth medium (EGM-2 BulletKit, Lonza, Basel, Switzerland) under standard conditions (37 °C and 5% CO_2_). The cells were seeded into 96-well plates at a density of 1.5 × 10^4^ cells/mL for the cell proliferation and cytotoxicity assays, and into 35 mm dishes for the cell migration assay. The cells were allowed to reach 70–80% confluence. Following a 12 h serum starvation period, for each in vitro assay, cells were allocated to three groups: non-irradiated control, 20 s laser, and 30 s laser.

The laser groups were subjected to a single irradiation without a hand piece and contact tip at an irradiation distance of 16 cm. In the 96-well plates, the actual energy level that the well received was 12.0 mJ/pulse, energy density was 2.95 mJ/cm^2^ per pulse, total energy was 2.39 J (20 s) and 3.59 J (30 s), and the total energy density was 0.59 J/cm^2^ (20 s) and 0.88 J/cm^2^ (30 s). In the 35 mm dishes, the actual energy level was 28.7 mJ/pulse, energy density was 2.98 mJ/cm^2^ per pulse, total energy was 5.74 J (20 s) and 8.61 J (30 s), and the total energy density was 0.60 J/cm^2^ (20 s) and 0.89 J/cm^2^ (30 s). Prior to laser irradiation, the culture medium in both the 96-well plates and 35 mm dishes was carefully removed.

#### 2.2.2. Cell Proliferation Assay

Cells were seeded into 96-well plates at a density of 1.5 × 10^4^ cells/mL and cultured under standard conditions for 48 h to allow stable cell attachment. Subsequently, the culture medium was replaced with low-serum medium containing 0.1% fetal bovine serum (FBS), and the cells were subjected to a 12 h serum starvation period to synchronize cellular activity prior to laser irradiation.

Immediately before laser irradiation, the culture medium was completely aspirated from each well to avoid interference with laser energy transmission. The tissue culture plates were uncovered during irradiation. During each irradiation session, four wells in the 96-well plate were irradiated simultaneously under a single laser beam to ensure uniform exposure conditions. Following laser exposure, fresh complete culture medium was promptly added to each well, and the cells were returned to the incubator and cultured under normal conditions for an additional 24 h.

Cell proliferation was assessed using a tetrazolium salt colorimetric assay (CellTiter 96 AQueous One Solution Cell Proliferation Assay, Promega, Madison, WI, USA) according to the manufacturer’s instructions. Briefly, 20 μL of CellTiter reagent was added to each well containing 100 μL of culture medium and incubated at 37 °C for 2 h. Absorbance was measured at 450 nm using a microplate reader (SpectraMax, Molecular Devices, Sunnyvale, CA, USA). Cell proliferation was expressed in a percentage relative to the control group. Eight technical replicates were set for each experimental condition (*n* = 8), and all experiments were independently repeated three times.

#### 2.2.3. Cytotoxicity Assay

Cytotoxicity was assessed using a lactate dehydrogenase (LDH) assay kit (Cytotoxicity Detection Kit: Roche, Mannheim, Germany). Cell seeding, serum starvation (0.1% FBS for 12 h), laser irradiation, and post-irradiation incubation were performed following the same workflow as described for the cell proliferation assay.

At 24 h after laser irradiation, culture supernatants were collected from each well. The LDH reaction mixture was freshly prepared according to the manufacturer’s instructions and immediately added to the collected supernatants. The samples were incubated for 30 min at room temperature away from light. Absorbance was measured at 490 nm using a microplate reader (SpectraMax, Molecular Devices, Sunnyvale, CA, USA). Relative cytotoxicity was calculated by normalizing the LDH activity in the laser-irradiated samples to that of the non-irradiated controls. Eight technical replicates were set for each experimental condition (*n* = 8), and all experiments were independently repeated three times.

#### 2.2.4. In Vitro Wound Healing Assay

Cell migration was evaluated using a scratch assay. Confluent HUVEC monolayers were prepared as follows: the cells were cultured in EGM-2 medium (EGM-2 BulletKit, Lonza, Basel, Switzerland) on 0.1% gelatin-coated 35 mm dishes (30 min, room temperature) and seeded at 3.0 × 10^5^ cells/well. Each dish was used with or without a single laser irradiation condition, with a total of 18 dishes in the control, 20 s laser and 30 s laser groups. After reaching 90–100% confluence (~24 h), the cells were serum starved in 0.1% fetal bovine serum EGM-2 for 24 h to minimize proliferation-related closure.

A uniform scratch was created using a sterile 1000 µL pipette tip at a constant angle, and the detached cells were removed by two PBS washes. Immediately before laser irradiation, the culture medium was completely aspirated, and irradiation was performed with the dish uncovered to avoid interference with laser energy transmission. Subsequently, fresh medium was added. The images were captured at 0, 6, 12, 18, and 24 h after irradiation using an inverted microscope (Eclipse Ti2, Nikon, Tokyo, Japan). All images were captured under identical imaging conditions using the same microscope and camera settings to ensure consistency across experimental groups. Wound width was quantified using ImageJ software (version 1.54p, NIH, Bethesda, MD, USA), and the migration rate was calculated as the percentage of wound closure relative to 0 h. Each condition was performed in six wells, and three random fields per well were analyzed. The area under the percent migration curve was calculated.

### 2.3. In Vivo Studies

#### 2.3.1. Creation of Palatal Wounds in Animals

**Figure 1:**
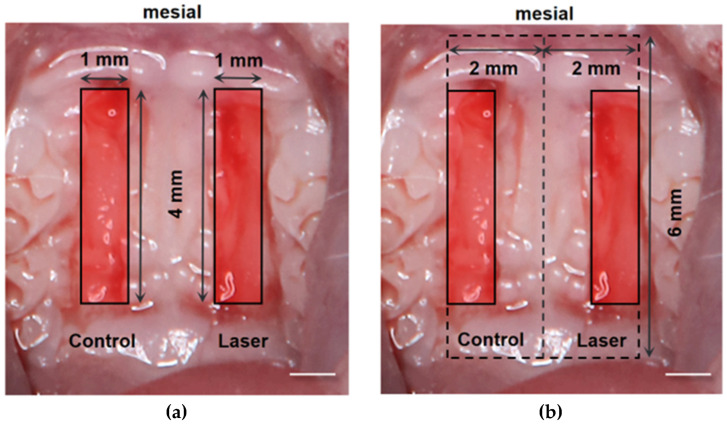
Experimental design of the murine palatal wound model: (**a**) standardized bilateral palatal wounds (4 × 1 mm) were created adjacent to the maxillary molars using a No. 11 scalpel. (**b**) Schematic representation of tissue sampling area at 48 h (dotted line). Gingival tissues, including the wound and 1 mm surrounding margins, were harvested for quantitative polymerase chain reaction analysis. Scale bar is 1 mm.

All experimental procedures were approved by the Institutional Animal Care and Use Committee of the Institute of Science Tokyo (approval no. A2025-033A). Fourteen male C57BL/6 mice (6–8 weeks old) were housed under standard laboratory conditions (12 h light/dark cycle) with free access to food and water. Mice were anesthetized with a three-drug combination consisting of medetomidine (0.75 mg/kg), midazolam (4 mg/kg), and butorphanol (5 mg/kg). The anesthetic mixture was administered intraperitoneally at a volume adjusted according to body weight. Bilateral full-thickness palatal excisional wounds (4 × 1 mm; depth, 1 mm) were created adjacent to the maxillary molars and parallel to the gingival margin using a double-bladed scalpel with a 1 mm interblade distance [28] (Figure 1).

#### 2.3.2. Laser Treatment

In each mouse, the wounds were assigned to the laser or non-irradiation control groups in a split-mouth design. LLLI was applied for 20 s in a defocused mode from a 10 mm distance with a contact chip (C800F, Morita, Osaka, Japan) and without water spray, inducing blood surface coagulation within the defects. During irradiation, the laser tip was maintained above the wound surface and moved in a uniform pattern confined to the wound margins to achieve homogeneous energy delivery across the defect. The spot diameter of each laser pulse at this distance was approximately 3.7 mm, yielding an actual energy level of 22.3 mJ/pulse, energy density of 0.21 J/cm^2^ per pulse, total energy of 0.45 J, and the total energy density of 11.2 J/cm^2^.

#### 2.3.3. Evaluation of Wound Healing

Standardized digital photographs of the palatal wounds were taken immediately after surgery and at 48 h postoperatively. Mice were anesthetized, the maxilla was retracted to fully expose the palate, and images were captured perpendicular to the wound plane using the same camera (fixed focal length) under identical lighting. A sterile millimeter ruler placed adjacent to the wound served as an internal calibration scale.

The wound area was quantified by digitally tracing the margins of the non-epithelialized area using ImageJ software. Each image was calibrated to the internal scale, and wound margins were delineated independently by two blinded examiners (K.M. and A.A.) using the polygon selection tool. The irradiated and control sides were compared within the same animal. The enclosed area was measured (mm^2^), and the reduction in wound area rate (%) at 48 h was calculated for each animal using the standard formula:

$$Reduction\;rate\;of\;wound\;area\;(\%)=\frac{0\;h\;area\;(mm^{2})-48\;h\;area\;(mm^{2})}{0\;h\;area\;(mm^{2})}\times 100\%$$

#### 2.3.4. mRNA Expression

At 48 h after irradiation, gingival tissue samples, including the wound and 1 mm surrounding margin, were collected (*n* = 14) (Figure 1b). The samples were homogenized using Lysing Matrix A (MP Biomedicals, Santa Ana, CA, USA). Total RNA was extracted with the RNeasy Fibrous Tissue Kit (Qiagen, Hilden, Germany), and RNA purity was assessed with a NanoDrop Lite spectrophotometer (Thermo Fisher Scientific, Waltham, MA, USA). Complementary DNA (cDNA) was synthesized using the PrimeScript RT Reagent Kit (Takara, Shiga, Japan).

Real-time quantitative polymerase chain reaction (qPCR) was performed on a Thermal Cycler Dice Real Time System II (Takara) using SYBR Premix Ex Taq II (Takara). The expression levels of vascular endothelial growth factor (VEGF), transforming growth factor-beta1 (TGF-β1), fibroblast growth factor-2 (FGF-2), interleukin-6 (IL-6), and tumor necrosis factor alpha (TNF-α) were normalized to glyceraldehyde 3-phosphate dehydrogenase. The primer sequences are provided in Table 1.

### 2.4. Statistical Analysis

Data are expressed as mean ± standard error (SE). The differences between two groups were analyzed using the paired Student’s *t*-test, while comparisons among multiple groups were assessed using one-way analysis of variance followed by the Tukey–Kramer post hoc test. In addition to the *p* values, effect sizes for in vivo comparisons were reported as mean differences together with their corresponding 95% confidence intervals (CIs) to support the main conclusions. A *p* value < 0.05 was considered statistically significant.

## 3. Results

### 3.1. In Vitro Study Results

#### 3.1.1. Cell Proliferation and Cytotoxicity Assays Results

**Figure 2:**
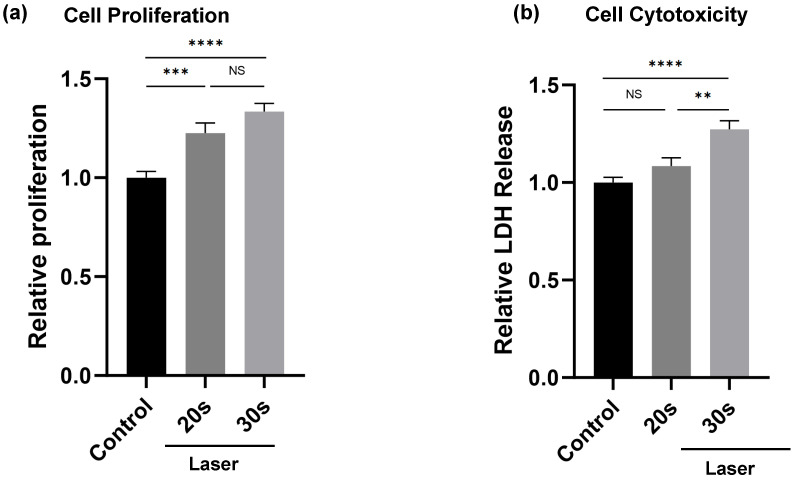
Representative result of 24 h cell proliferation and cytotoxicity assays after laser irradiation. HUVECs were irradiated with Er:YAG laser for 20 s or 30 s and analyzed after 24 h. (**a**) Both 20 s and 30 s irradiations significantly increased the cell proliferation compared with the control group, with no significant difference between the two laser groups. (**b**) Lactate dehydrogenase (LDH) release slightly increased after 30 s irradiation, indicating mild cellular stress, whereas no significant change was observed after 20 s irradiation. Data are presented as the mean ± SE (*n* = 8). ****: *p* < 0.0001, ***: *p* < 0.001, **: *p* < 0.01, NS: not significant.

At 24 h post-irradiation, both 20 s and 30 s exposure laser groups showed significantly increased cell proliferation compared to the control (*p* < 0.001 and *p* < 0.0001, respectively), as evidenced by relative proliferation values in the cell viability assay. No significant difference was observed between the 20 s and 30 s laser groups (Figure 2a).

In contrast, LDH cytotoxicity assay results demonstrated a significant increase in cytotoxicity only in the 30 s laser group compared to the control (*p* < 0.01), while the 20 s laser group showed a slight increase, but it did not reach statistical significance (Figure 2b). The experiments were independently repeated thrice with consistent results.

#### 3.1.2. In Vitro Wound Healing Assay Results

**Figure 3:**
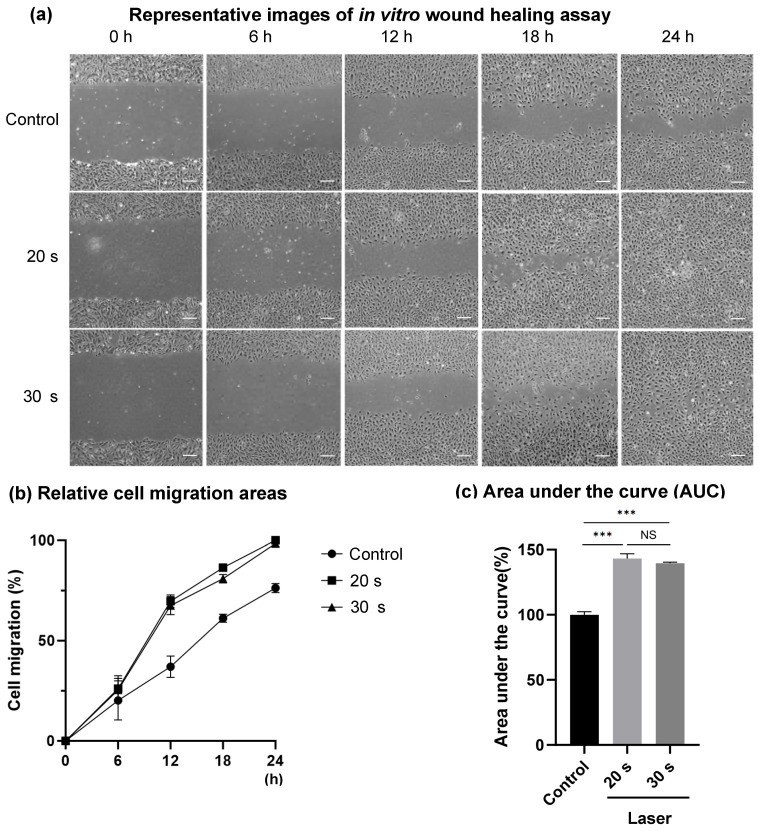
(**a**) Representative images of the in vitro wound healing assay. Images were taken immediately after irradiation (0 h), and after 6, 12, 18, and 24 h of incubation. Compared with the control, the laser groups, particularly the 20 s laser group, showed enhanced cell migration. (**b**,**c**) Quantitative evaluation of cell migration was performed following Er:YAG laser irradiation for 20 and 30 s. Both laser-irradiated groups exhibited significantly enhanced migratory activity compared with the control group. No significant difference was observed between the 20 s and 30 s irradiation groups. Data are presented as the mean ± SE (*n* = 6). ***: *p* < 0.001, NS: not significant. Representative images shown at 40× magnification. Scale bar is 100 μm.

Microscopic images from the in vitro wound healing assay revealed faster wound closure in both the laser-treated groups compared to the control across all time points (0–24 h), with nearly complete gap closure observed by 24 h in the 20 s and 30 s laser groups (Figure 3a).

Quantitative analysis confirmed this observation: the percentage of wound closure was significantly higher in both the laser-irradiated groups than in the control group at each time point. Furthermore, the area under the curve (AUC) for cell migration showed significantly elevated values in the 20 s and 30 s laser groups compared to the control (*p* < 0.001); however, no significant difference was detected between the 20 s and 30 s laser groups (Figure 3b,c). This suggested that both laser durations effectively promoted cellular migration, with 20 s potentially being sufficient to elicit maximal response.

### 3.2. In Vivo Study Results

#### 3.2.1. General Observations Results

All animals survived the surgical procedures and postoperative period without complications. During surgery, no abnormal bleeding was observed in either group. Following irradiation, the laser-treated wounds showed mild coagulation and whitening of the surface layer of blood without any evidence of major thermal damage, such as carbonization. The wounds in both groups exhibited uneventful healing without infection, and all samples were successfully harvested.

#### 3.2.2. Wound Healing Analysis Results

**Figure 4:**
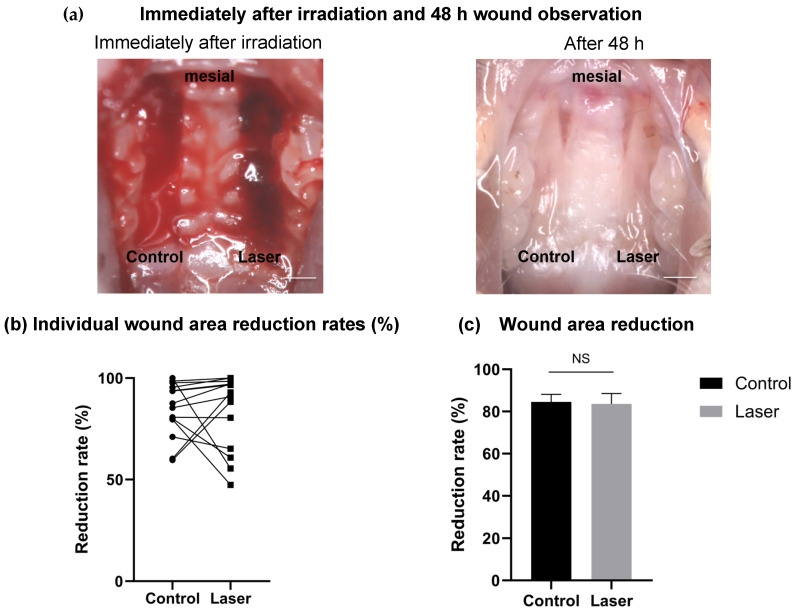
(**a**) Representative intraoral photographs of palatal mucosal wounds at baseline and after 48 h. “Control” and “Laser” indicate the non-irradiation control and laser-irradiated sites, respectively. Both sites show progressive wound closure after 48 h. (**b**) Individual reduction rates (%) of wound area between the groups. (**c**) The mean wound area reduction rate was comparable between the groups. Data are presented as the mean ± SE (*n* = 14). NS: not significant. Scale bar is 1 mm.

Representative images of palatal wounds at baseline and 48 h post-surgery are shown in Figure 4a. Both the laser and control groups exhibited marked contraction and epithelial coverage over time. Quantitative analysis revealed that the mean wound reduction rate at 48 h was comparable in the laser and control groups (control group: 84.5 ± 3.6%, laser group: 83.6 ± 4.9%, *p* = 0.87, *n* = 14) (Figure 4b,c). The mean difference (laser–control) was −0.88%, with a 95% confidence interval ranging from −12.72% to +10.95%.

#### 3.2.3. mRNA Expression Analysis Results

**Figure 5:**
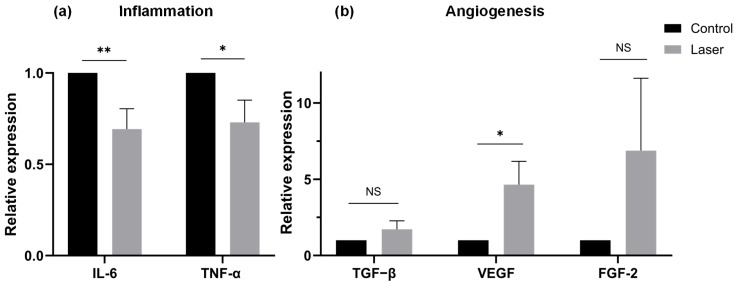
(**a**) mRNA expression of inflammatory cytokines in the palatal mucosal wounds after 48 h: IL-6 and TNF-α. Both cytokines were significantly downregulated in the laser group compared with controls. (**b**) mRNA expression of angiogenic cytokines: TGF-β1, VEGF, and FGF-2. VEGF expression was significantly upregulated in the laser group, while no significant differences were observed for TGF-β1 or FGF-2, although they showed higher values on average. IL-6, interleukin-6; TNF-α, tumor necrosis factor alpha; TGF-β1, transforming growth factor beta; VEGF, vascular endothelial growth factor; FGF-2, fibroblast growth factor-2. Data are presented as mean ± SE (*n* = 14). *: *p* < 0.05, **: *p* < 0.01, NS: not significant.

Real-time qPCR analysis demonstrated that the expressions of IL-6 and TNF-α were significantly downregulated compared with controls (IL-6: *p* = 0.008; TNF-α: *p* = 0.04). VEGF expression was significantly upregulated (*p* = 0.03). Although TGF-β1 and FGF-2 showed higher values on average, the differences were not significant (FGF-2 [*p* = 0.24] and TGF-β1 [*p* = 0.22]) (Figure 5). The mean differences (laser–control) were −0.307 (95% CI −0.549 to −0.064) for IL-6, −0.270 (95% CI −0.536 to −0.005) for TNF-α, 0.721 (95% CI −0.500 to 1.942) for TGF-β, 3.648 (95% CI 0.320 to 6.976) for VEGF, and 5.879 (95% CI −4.451 to 16.210) for FGF-2.

## 4. Discussion

This study demonstrated that low-level Er:YAG laser irradiation directly enhanced endothelial cell migration in vitro while modulating early gingival wound healing in vivo through coordinated regulation of inflammatory and angiogenic responses. These findings support the notion that Er:YAG-based LLLI exerts biologically relevant effects beyond tissue ablation and may contribute to early wound repair by influencing the molecular and cellular wound microenvironment [1,2].

In the in vitro wound healing assay, low-level Er:YAG laser irradiation markedly enhanced endothelial cell migration compared with the control group. Endothelial cell migration may be associated with angiogenic processes in vivo, governing sprout initiation, directional guidance, and capillary network formation [29,30]. Effective angiogenesis requires a finely tuned microenvironment in which endothelial migration and proliferation are temporally coordinated rather than simultaneously maximized [31]. Consistent with these principles, previous PBM and LLLI studies have demonstrated enhanced endothelial motility following laser irradiation, suggesting that laser-induced modulation of cytoskeletal and adhesion-related signaling contributes to angiogenic activation [27]. Enhanced endothelial migration may therefore promote neovascularization and indirectly facilitate subsequent epithelial coverage and tissue maturation in vivo by accelerating capillary ingrowth, extracellular matrix remodeling, and epithelial–endothelial interactions [29,31].

In the in vivo experiment, wound closure in the laser-treated group was not significantly accelerated compared with untreated controls at the 48 h time point. The irradiation parameters used in this study were selected based on distinct experimental objectives for the in vitro and in vivo models. For the in vitro experiments, a defocused distance of 16 cm was chosen to obtain a laser spot size approximately equivalent to the surface area of a 35 mm culture dish, allowing uniform low-level irradiation of the entire cell monolayer. Conversely, the in vivo irradiation protocol was designed to reflect clinically applicable Er:YAG laser procedures, in which low-level irradiation is typically performed within a defocus range of 5 to 10 mm distance using a contact tip. Accordingly, a 10 mm defocused distance was selected to ensure clinical relevance and safe modulation of the wound surface.

The discrepancy between the pronounced in vitro responses and the absence of enhanced wound closure in vivo at 48 h can be attributed to fundamental differences in the irradiation conditions between the two experimental settings. In vitro irradiation is applied directly to a monolayer of cells, allowing efficient delivery of laser energy to the target cells. However, in vivo laser energy is subject to scattering and absorption across multiple tissue layers within the wound and surrounding gingival tissues, resulting in substantially lower effective energy reaching resident cells. In addition, in the present in vivo model, low-level Er:YAG laser irradiation was applied broadly to the wound surface rather than being directed specifically to the vascular endothelial cells. As a result, direct stimulation of angiogenic cellular responses was likely limited at this early stage. These factors may explain why robust cellular responses were observed in vitro, whereas macroscopic wound closure was not significantly accelerated at 48 h in vivo. Longer observation periods and further optimization of irradiation parameters tailored to in vivo conditions may therefore be required to fully elucidate the therapeutic potential of Er:YAG-based low-level laser irradiation in gingival wound healing.

Nevertheless, VEGF expression was significantly upregulated in laser-irradiated gingival wounds. The VEGF/VEGFR signaling axis is a central regulatory pathway in both physiological and pathological angiogenesis and is widely recognized as a master regulator of vascular responses [10]. Increased VEGF expression is associated with accelerated granulation tissue formation and improved wound healing outcomes in both cutaneous and oral wound models [32]. The concurrent observation of VEGF upregulation in vivo and enhanced endothelial migration in vitro in this study supports the interpretation that Er:YAG-based LLLI activates angiogenesis-related signaling during the early phase of gingival wound repair [25]. At the molecular level, several mechanisms may underlie the angiogenic effects observed following Er:YAG-based LLLI. LLLI stimulates mitochondrial activity, leading to transient increases in ATP production and reactive oxygen species, which act as secondary messengers to activate intracellular signaling cascades, including phosphoinositide 3-kinase/protein kinase B (PI3K/Akt) and mitogen-activated protein kinase/extracellular signal-regulated kinase (MAPK/ERK) pathways [3]. Activation of these pathways has been linked to transcriptional upregulation of VEGF and other pro-angiogenic mediators. Recent evidence also indicates that VEGF-driven angiogenesis can be reinforced by epigenetic and transcriptional feedback mechanisms in endothelial cells, further amplifying angiogenic responses once initiated [33]. Although Er:YAG laser energy is primarily absorbed by water, non-ablative irradiation may induce photophysical or mechanical perturbations at the cell membrane, leading to activation of the mechanosensitive signaling pathways involved in angiogenic and inflammatory regulation [3,34].

Nevertheless, FGF-2 and TGF-β1 expression levels tended to increase following Er:YAG irradiation; these changes did not reach statistical significance at 48 h. Both growth factors are primarily involved in the proliferative and remodeling phases of wound healing. TGF-β1 critically regulates fibroblast proliferation, extracellular matrix deposition, immune modulation, and scar formation [35]. In oral mucosal wounds, TGF-β1 expression exhibits site-specific temporal patterns distinct from those observed in cutaneous wounds, often peaking after the initial inflammatory phase [36]. Similarly, basic fibroblast growth factor-2 (FGF-2) is a key mediator of angiogenesis and epithelial proliferation and enhances wound healing in healing-impaired models, typically during later stages of repair [37]. Therefore, despite the increased values obtained on average, the absence of significant changes in this study may be due to the early evaluation time point, which corresponds primarily to the inflammatory phase. Extending the observation period to include the proliferative phase would help clarify the temporal dynamics of these molecular responses to Er:YAG irradiation.

This study also provides in vivo evidence that low-level Er:YAG laser irradiation modulates transcriptional regulation of major pro-inflammatory mediators during gingival wound healing. IL-6 and TNF-α are central cytokines in acute inflammation, contributing to leukocyte recruitment, vascular permeability, and extracellular matrix degradation [38,39]. Excessive or prolonged activity of these cytokines is associated with delayed epithelial closure and impaired connective tissue repair in oral and periodontal wounds [7]. Previous in vitro studies have shown that Er:YAG laser irradiation suppresses pro-inflammatory cytokine expression and inflammasome activation in *Porphyromonas gingivalis* lipopolysaccharide-stimulated periodontal ligament fibroblasts, providing mechanistic support for an anti-inflammatory effect [22]. Laser-mediated anti-inflammatory effects have been associated with modulation of NF-κB signaling, a central regulator of inflammation and angiogenesis during tissue repair [24,40]. In addition, photobiomodulation influences immune cell behavior, including macrophage polarization toward pro-reparative phenotypes characterized by reduced pro-inflammatory cytokine production and enhanced secretion of angiogenic mediators such as VEGF [23,41].

Although low-level Er:YAG laser irradiation induced a favorable cytokine profile and upregulation of angiogenic markers, these molecular changes did not result in accelerated wound closure at 48 h. Suppression of inflammation alone is insufficient to immediately drive macroscopic wound closure; closure requires coordinated epithelial migration, stromal remodeling, and tissue contraction, which typically occur after the early inflammatory phase. Similarly, while VEGF upregulation reflects early endothelial activation, structural angiogenesis and functional microvascular formation develop later, preceding subsequent rather than immediate wound area reduction [4,8,41,42,43].

Clinically, low-level Er:YAG laser irradiation is frequently applied to the blood clot surface during periodontal pocket treatment and regenerative surgical procedures. A randomized controlled clinical trial demonstrated that Er:YAG laser-assisted comprehensive periodontal pocket therapy using blood clot surface coagulation at the pocket entrance provided significant clinical benefits for residual periodontal pockets, supporting the therapeutic relevance of Er:YAG laser application beyond mechanical debridement [44]. Moreover, a case series of Er:YAG laser-assisted periodontal regenerative therapy and bone regenerative therapy using blood clot surface coagulation have reported favorable healing and regenerative outcomes, suggesting that modulation of the early wound and blood clot environment may contribute to improved tissue regeneration [45,46]. Furthermore, a randomized controlled trial showed that Er:YAG- and Nd:YAG-based LLLI combined with medical collagen improved third molar extraction wound healing, supporting the broader clinical applicability of laser-based LLLI in oral wound management [47]. Another limitation is the absence of findings suggesting that Er:YAG-based LLLI not only exhibits physical effects to enhance the stability of blood clots but also exhibits biological effects by modulating the early inflammatory and angiogenic microenvironment. Such early biological regulation may be particularly relevant in periodontal surgery, where stabilization of the wound site and blood clot is critical for subsequent tissue regeneration.

This study has some limitations. Only selected inflammatory and angiogenic mediators were evaluated at the mRNA level, and broader gene panels or transcriptomic approaches could provide a more comprehensive understanding of Er:YAG laser-induced molecular changes. In particular, future studies may incorporate markers related to extracellular matrix remodeling (e.g., MMP-2, MMP-9, and collagen I/III), growth factor-mediated angiogenic signaling (e.g., PDGF and Ang-1/Ang-2), and epithelial responses, including keratinocyte differentiation and migration markers such as K14 and K17, to further delineate the molecular events underlying gingival wound healing. Moreover, while in vitro assays are valuable for characterizing specific cellular responses, they cannot fully recapitulate the complexity of the in vivo wound environment, which involves immune cell interactions, extracellular matrix remodeling, and biomechanical cues [41]. Another limitation of this study is the absence of histological evaluation. Because early stage murine palatal tissues are fragile and limited in size, intact sections suitable for histological analysis could not be obtained, which restricted morphological interpretation of the wound healing process. In addition, the use of a single early time point and a murine palatal wound model may limit extrapolation to the temporal dynamics and tissue characteristics of human gingival healing. Future studies using larger animal models and extended observation periods may allow closer integration of the molecular findings with tissue-level changes.

Despite these constraints, the present findings provide a foundation for the therapeutic application of low-level Er:YAG laser irradiation in gingival wound management. Further studies incorporating temporal gene expression profiling, histological analysis, and functional vascular assessments should delineate the underlying mechanisms and confirm clinical relevance in larger animal models and controlled clinical trials [1,2,17].

## 5. Conclusions

Low-level Er:YAG laser irradiation enhances endothelial cell proliferation and migration, and modulates anti-inflammatory and pro-angiogenic effects in gingival wounds, suggesting its clinical potential to promote gingival wound healing.

## Figures and Tables

**Table 1:** Primer sequences used in this study.

Gene	Forward (5′-3′)	Reverse (3′-5′)
*G* *apdh*	AGGACCAGGTTGTCTCCTGT	TTACTCCTTGGAGGCCATGT
*I* *l* *-6*	TGAACAACGATGATGCACTTGC	TCCAGTTTGGTAGCATCCATCA
*T* *nf* *-α*	GTGCCTATGTCTCAGCCTCTT	CGATCACCCCGAAGTTCAGTA
*T* *gf* *-β1*	TGAACCGGCCTTTCCTGCTTCTCATG	GCGGAAGTCAATGTACAGCTGCCGC
*V* *egf*	ACCCCGACGAGATAGAGTACA	TCCAGGGCTTCATCGTTACAG
*F* *gf* *-2*	CCAAGCAGAAGAGAGAGGAGT	CACTACTCACAGAAGCCAGCA

## Data Availability

The data presented in this study are available on request from the corresponding author.
